# A genetically encoded L-rhamnose biosensor for monitoring marine polysaccharide depolymerization

**DOI:** 10.1007/s00253-026-13724-1

**Published:** 2026-01-29

**Authors:** Yannick L. Wolf, Thomas Bayer, Uwe T. Bornscheuer

**Affiliations:** Department of Biotechnology and Enzyme Catalysis, Institute of Biochemistry, University of Greifswald, Greifswald, Germany

**Keywords:** Biosensor, L-rhamnose, Marine carbohydrates, Polysaccharide degradation, Biomass valorization

## Abstract

**Abstract:**

Marine macroalgae, particularly their complex polysaccharides, are an untapped renewable source of high-quality monosaccharides and related building blocks. To utilize this feedstock for industrial applications, the enzymatic depolymerization by marine microorganisms has been shown to be effective. A prime example is the common green alga *Ulva*, with its storage polysaccharide ulvan, which contains high quantities of L-rhamnose and D-glucuronic acid. As suitable high-throughput methods for analyzing the enzymatic degradation of complex polysaccharides are still lacking, a transcription factor–based biosensor is described here that utilizes the P_rha_BAD promoter native to *E. coli*, which is specific for L-rhamnose. This biosensor exhibited a linear response, enabling the quantification of L-rhamnose within a concentration range of 10–1000 µM. The introduction of a T7 stem-loop improved the performance, and various fluorescent reporter genes were studied. The optimized system was then used to evaluate various stages of the ulvan degradation cascade in terms of L-rhamnose release, confirming its applicability to complex sugar mixtures. A detectable fluorescence signal was only generated when all the necessary enzymes for breaking down the polymer into undecorated monosaccharides were present, highlighting the biosensor’s specificity. The application of this method to the degradation of *Ulva* sp. biomass samples of various origins was also successfully demonstrated. This establishes the biosensor as a promising method for further high-throughput investigations.

**Key points:**

• *Development of an improved transcription factor-based biosensor for L-rhamnose.*

• *Biosensor application for the analysis of enzymatic polysaccharide degradation.*

• *Reliable quantification of L-rhamnose in complex carbohydrate mixtures.*

**Graphical Abstract:**

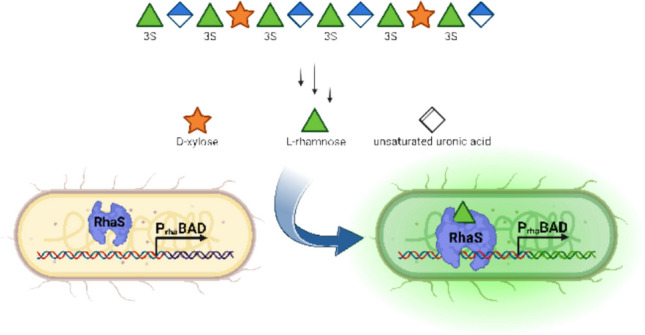

**Supplementary Information:**

The online version contains supplementary material available at 10.1007/s00253-026-13724-1.

## Introduction

Rising temperatures and increasing nutrient input into the world’s oceans are contributing to both the frequency and severity of algal blooms. In particular, the benthic green macroalgae of the genus *Ulva* lead to so-called green tides, resulting from rapid algal growth and detachment from the seabed. The negative consequences of this phenomenon are significant, affecting different economic sectors, including fisheries and tourism (Ménesguen and Piriou 1995; Smetacek and Zingone 2013). To address these issues, the costly removal of algal waste is employed. However, the accumulated biomass may serve as a source of metabolites and valuable building blocks with applications in the food, pharmaceutical, and biorefinery industries (Shah et al. 2023; Gajanayaka et al. 2025).

One of the major components of the biomass of *Ulva* macroalgae is ulvan, a complex and highly sulfated polysaccharide. This cell wall component accounts for up to 30% of algal dry weight (Lahaye and Robic 2007). The structural composition of ulvan reveals a predominant disaccharide repeating unit of D-glucuronic acid (GlcA) or L-iduronic acid (IdoA), which is β-1,4-linked to L-rhamnose-3-sulfate (Rha3S). This major building block is α-1,4-linked to the next disaccharide unit in the main chain. Depending on the ulvan source, some of the uronic acids may be replaced by D-xylose (Xyl) or D-xylose-2-sulfate (Xyl2S) (Lahaye et al. 1997; Lahaye 1998). Hence, the corresponding monosaccharides can be obtained from ulvan degradation and converted into fine chemicals or used as carbon feedstock in fermentation (Cesário et al. 2018; Dutschei et al. 2022). Enzymatic depolymerization is advantageous over chemical degradation processes because it does not require harsh reaction conditions and auxiliary chemicals. Due to the mild enzymatic depolymerization conditions, monosaccharides can be accessed in high quality. Specialized marine microorganisms such as the Flavobacterium *Formosa* *agariphila* KMM 3901^T^ (*F. agariphila*), which originates from the North Sea, harbor ulvanolytic enzymes that allow them to utilize algal polysaccharides as feedstocks (Collén et al. 2011; Kopel et al. 2016; Salinas and French 2017; Reisky et al. 2018). The genes encoding these enzymes are often organized in clusters called polysaccharide utilization loci (PULs) (Terrapon et al. 2015). In *F.* *agariphila*, at least 13 different PULs are known, which have been designated A to M according to their occurrence in the genome, with the genes for ulvan depolymerization located in PUL H (Mann et al. 2013; Salinas and French 2017). Owing to the complexity of ulvan, a series of enzymes must work in tandem to break down the polysaccharide and metabolize monosaccharides (Reisky et al. 2019). In this context, the degree of depolymerization is of crucial importance for further processing. For industrial applications, therefore, the hydrolytic activities of multiple enzymes and the degree of carbohydrate depolymerization have to be assessed.

Until now, the degradation of polysaccharides has commonly been monitored by reducing-end assays for quantification, using compounds such as dinitrosalicylic acid (Miller 1959) or 3-methyl-2-benzothiazolinone hydrazone (Anthon and Barrett 2002) that yield a colorimetric read-out. For qualitative analysis, electrophoretic methods like fluorophore-assisted carbohydrate electrophoresis (FACE) (Starr et al. 1996) or carbohydrate polyacrylamide gel electrophoresis (C-PAGE) are frequently employed (Reisky et al. 2019). However, quantitative reducing-end assays do not provide specific information about the products formed. In contrast, gel electrophoresis may enable the differentiation of oligomers and monosaccharides. Nonetheless, both strategies are time-consuming and only allow low-throughput analysis. To address these and related limitations, biosensors have been used as simple, robust, and cost-effective detection tools for various metabolites (Choe and Titov 2022; Bayer et al. 2023; Pisani and Carbonell 2025). Genetically encoded biosensors generally consist of two functionally linked components: a sensing and a transduction unit (Xia et al. 2019). The sensing unit can be protein-based (e.g., transcription factors, two-component systems, or fluorescent probes) or RNA-based (e.g., riboswitches and toehold switches) (Bayer et al. 2023; Pisani and Carbonell 2025). The sensing unit controls the expression of the transduction module in the form of reporter genes in the presence of the target analyte. The transduction module then converts the detected input into a physical output signal, such as fluorescence or bioluminescence of reporter gene products. Due to their high specificity and suitability for high-throughput analysis, biosensor systems have enabled the detection and quantification of natural metabolites as well as xenobiotics, the screening of enzyme libraries (Markel et al. 2020; Sakoleva et al. 2025), or the engineering of metabolic pathways and reaction conditions (Hossain et al. 2020; Yi et al. 2021; Verma et al. 2022; Bayer et al. 2023).

In this work, we aimed at implementing a genetically encoded biosensor for the detection of L-rhamnose (Rha), one of the main products of ulvan degradation. Monitoring of this monosaccharide can be facilitated by the regulatory elements of the L-rhamnose metabolization operon of *Escherichia coli* (*E. coli*), in which the expression of the *rhaBAD* genes is controlled by the P_rha_BAD promoter and the activator proteins RhaR and RhaS (Egan and Schleif 1993). The latter two are members of the AraC-XylS family of positively regulated expression systems. The binding of Rha to RhaR, which is constitutively produced at basal levels, enhances *rhaR* and *rhaS* transcription. Ultimately, the binding of Rha to RhaS stimulates the expression of the *rhaBAD* genes in vivo (Egan and Schleif 1993; Bhende and Egan 1999; Wickstrum et al. 2007; Kolin et al. 2008). Unlike the “all-or-nothing” response of some established promoter systems, such as the natural lactose- and arabinose-inducible P_lac_ and the P_ara_BAD promoters, respectively, the expression from P_rha_BAD increases proportionally with Rha (i.e., inducer) concentration (Khlebnikov et al. 2002; Bayer et al. 2015; Živič et al. 2025). This makes the P_rha_BAD promoter well-suited for biosensing applications. Consequently, we constructed and evaluated various genetic modifications of the Rha-sensing P_rha_BAD promoter, driving the expression of reporter genes encoding different fluorescent proteins. The optimized biosensor system not only enabled the reliable detection of Rha over a broad concentration range (10–1000 µM); it was successfully used to analyze the gradual degradation of the marine polysaccharide ulvan by PUL H-encoded enzymes from *F. agariphila*. The applicability of the biosensor was further demonstrated by the Rha-based polysaccharide degradation directly from three algal biomass samples originating from different *Ulva* species.

## Materials and methods

All standard chemicals were obtained from commercial suppliers and used without further purification, including the following monosaccharides: D-glucose, D-galactose, and L-rhamnose (Sigma-Aldrich), D-fructose and D-mannose (Fluka), L-sorbose (Serva), L-arabinose and D-xylose (Carl Roth), as well as D-glucuronic acid (Merck). Two samples of *Ulva* sp. algae were collected: one from Helgoland in the North Sea (Germany, 54°11′16.8″N 07°52′12.0″E, September 2016) and one from Lubmin at the Baltic Sea (Germany, 54°09′01.0″N 13°38′44.8″E, July 2017). A third biomass sample was purchased from www.kulau.de (GTIN: 4260171055056, origin: Atlantic coast of Spain). The polysaccharide ulvan was extracted as described in the literature (Robic et al. 2009). The L-rhamnose assay kit was obtained from Megazyme/Neogen and used as instructed by the supplier. *E. coli* BL21(DE3) and *E. coli* TOP10 were purchased from Thermo Scientific/Invitrogen (Darmstadt, Germany).

### Construction of biosensor plasmids

The plasmid pCK302 (Kelly et al. 2016), which harbors the gene encoding the regulatory protein RhaS under constitutive expression and the reporter gene encoding the superfolder green fluorescent protein (sfGFP) (Pédelacq et al. 2006) downstream of P_rha_BAD, was ordered from Addgene (#87,768; https://www.addgene.org/87768/). Mutations in the region of the two RhaS binding sites and insertion of the stem-loop between the promoter and reporter gene were performed by using the Q5^®^ Site-Directed Mutagenesis Kit (New England Biolabs). The reporter genes (sfGFP, eGFP, mStayGold, and mCherry) were replaced in the pCK302sl backbone using Gibson Assembly^®^ Master Mix (New England Biolabs). Unless stated otherwise, polymerase chain reactions (PCRs) and processing of amplification products were performed as instructed by the supplier. All primers used in this work are listed in Table S1. Transformation of chemically competent *E. coli* TOP10 by heat-shock followed established protocols. Cloning success was confirmed by Sanger sequencing (Microsynth AG, Balgach, Switzerland).

### In vivo biosensing and fluorescence measurements

In preparation for biosensor applications, 4 mL of lysogeny broth (LB) medium containing 100 µg/mL ampicillin was inoculated with a single colony of *E. coli* BL21(DE3) carrying the desired biosensor plasmid and cultured at 37 °C with shaking (150 rpm) for 16 h. The optical density at 600 nm (OD_600_) of the preculture was determined. Using sterile ddH_2_O, 10× LB medium (250 g/L), ampicillin (100 mg/mL), and the preculture, a double-concentrated cell mixture was prepared, containing LB medium (50 g/L) supplemented with 200 µg/mL ampicillin (OD_600_ = 0.2). In a 96-well black/clear bottom plate (Greiner, Item No. 655096), 50 µL of the Rha-containing samples to be analyzed (see below) was loaded. Then, 50 µL of the 2× cell mixture was added to each well. After briefly mixing, both the OD_600_ and the fluorescence (*λ*_ex_: 485 nm; *λ*_em_: 520 nm) were measured (*t*_0_ = 0 h) on a microtiter plate reader (BioTek Synergy H1, Agilent Technologies, USA). For cultivation, the 96-well plate was sealed with an AeraSeal™ to prevent evaporation while allowing gas exchange and incubated at 37 °C with shaking (800 rpm). OD_600_ and fluorescence were determined every hour for up to 7-h cultivation time. For plasmids featuring different reporters (Table S1), the fluorescence was measured as follows: eGFP (*λ*_ex_ 485 nm; *λ*_em_ 520 nm), mStayGold (*λ*_ex_ 485 nm; *λ*_em_ 520 nm), and mCherry (*λ*_ex_ 580 nm; *λ*_em_ 610 nm).

### Recombinant enzyme production and purification

*E. coli* BL21(DE3) cells harboring pET28a-derived expression plasmids were used for recombinant protein production of the following PUL H enzymes from *F. agariphila*: P10_PL40, P24_GH3, P31_GH39, P32_S1_8, P33_GH105, and P36_S1_25 (Reisky et al. 2019). A preculture was prepared by inoculating 4 mL of LB medium supplemented with 50 µg/mL kanamycin with a single colony of the desired transformant and incubating at 37 °C with shaking at 150 rpm for 16 h. Subsequently, terrific broth (TB) medium containing 50 µg/mL kanamycin was inoculated with 1% (ν/ν) of the preculture. Cultures were grown at 37 °C with shaking (150 rpm) until an OD_600_ of 0.6–0.8 was reached. Enzyme production was induced with 0.5 mM isopropyl β-D-1-thiogalactopyranoside (IPTG) and performed at 20 °C for an additional 20 h. Cells were harvested by centrifugation (5000 × g, 4 °C for 30 min). The resulting cell pellets were resuspended in lysis buffer (50 mM Tris-HCl, 300 mM NaCl; pH 8.0), and cells were lysed by sonication on ice, using a Sonoplus HD2070 sonicator (Bandelin) with two cycles of 3 min at 50% amplitude. The insoluble cell debris was removed by centrifugation (10,000 × g, 4 °C for 30 min). The clarified lysate was submitted to ion metal affinity chromatography to purify recombinant proteins containing N-terminal 6xHis-tags, using ROTI^®^ Garose-His/Ni resin (Carl Roth; 1 mL bed volume). Protein binding was performed in 50 mM Tris-HCl buffer and 300 mM NaCl (pH 8.0), containing 20 mM imidazole. The resin was washed four times with this buffer (5 mL). Bound proteins were eluted with 50 mM Tris HCl buffer and 300 mM NaCl (pH 8.0), containing 300 mM imidazole (2.5 mL). Imidazole was removed and the buffer exchanged by desalting protein-containing fractions, using PD-10 columns (Cytiva) equilibrated with 35 mM Tris-HCl buffer, and 50 mM NaCl (pH 8.0). Protein concentrations were determined using the Pierce™ BCA Protein Assay Kit (Thermo Scientific) according to the manufacturer. The purified proteins were flash-frozen in liquid nitrogen and stored at − 20 °C until further use.

### Ulvan hydrolysis

Prior to the reaction, a 10-mg/mL stock solution of ulvan was prepared in 35 mM Tris-HCl buffer, and 50 mM NaCl (pH 8.0). The solution was incubated at 37 °C with vigorous shaking (800 rpm) until it became homogeneous and free of gel-like particles. The final reaction mixture contained 1 mg/mL ulvan and 0.1 µM of each of the purified PUL H enzymes (P10_PL40, P24_GH3, P31_GH39, P32_S1_8, P33_GH105, and P36_S1_25 as indicated) in 35 mM Tris-HCl buffer, 50 mM NaCl (pH 8.0). Samples were incubated at 20 °C and 800 rpm for 20 h. Enzymes were inactivated at 90 °C for 10 min. Finally, to remove denatured proteins, the samples were centrifuged at 15,000 × g for 10 min.

### Biomass hydrolysis

To investigate biomass degradation, *Ulva* algae were collected from the Baltic Sea (Lubmin, Germany) and the North Sea (Helgoland, Germany). A commercially available sample of dried *Ulva* algae was purchased from www.kulau.de as described above. The collected algae were washed several times with water to remove contaminants, such as sand and salts, and then air-dried for up to 48 h. To increase the surface area, all dry algal samples were ground using a blender and mortar. Algae (50 mg) were dispensed into 2-mL reaction tubes. For the negative control, only the reaction buffer (35 mM Tris-HCl, 50 mM NaCl; pH 8.0) was added. For the samples with enzymes, 0.2 µM of the PUL H enzymes (P10_PL40, P24_GH3, P31_GH39, P32_S1_8, P33_GH105, and P36_S1_25) was added. The reaction was performed at 25 °C with shaking (800 rpm). After 20-h reaction time, the biomass was removed by centrifugation (15,000 × g for 10 min). The resulting supernatant was transferred to a new 1.5-mL reaction tube. To stop the enzymatic reaction, these samples were heated to 90 °C for 10 min, followed by centrifugation at 15,000 × g for 10 min to remove denatured protein.

### Data analysis

To evaluate the collected biosensor data, the fluorescence value for each well was divided by the corresponding OD_600_ using Microsoft Excel 365. The fold-increase above background was calculated by dividing the obtained values by those of the corresponding sample not containing any L-rhamnose. Each measurement was performed at least in three independent replicates. Mean values and the standard deviation were calculated using Microsoft Excel 365 again.

## Results

For the biosensing module for Rha detection, the regulatory elements native to *E. coli* were employed. The previously assembled plasmid pCK302 harbors the *rhaS* gene constitutively produced from the P_ampR_ promoter and its native ribosome binding site (RBS). Noteworthy, the regulator RhaR is not required for upregulation of RhaS (Egan and Schleif 1993; Bhende and Egan 1999; Wickstrum et al. 2007; Kolin et al. 2008). This is important to circumvent catabolic repression of the native RhaS in *E. coli* in the presence of preferred monosaccharide feedstocks (e.g., D-glucose) in complex sugar mixtures as analyzed in this work (Hogema et al. 1998; Deutscher 2008; Ammar et al. 2018). For transduction, pCK302 contains sfGFP as the reporter, which is cloned downstream of the P_rha_BAD promoter region and a synthetic RBS (Kelly et al. 2016).

First, to determine whether the response of the promoter and reporter gene behaved linearly in *E. coli* BL21(DE3), the fluorescence from sfGFP was determined in vivo at different Rha concentrations (0–1 mM). Satisfactorily, the OD_600_-normalized fluorescence output increased linearly over the cultivation time of 6 h at 37 °C. However, the fold increase in fluorescence above background peaked after 4 h (Figure S1). At elongated cultivation times, the background fluorescence increased, yielding reduced fold increases over background (data not shown).

**Fig. 1:**
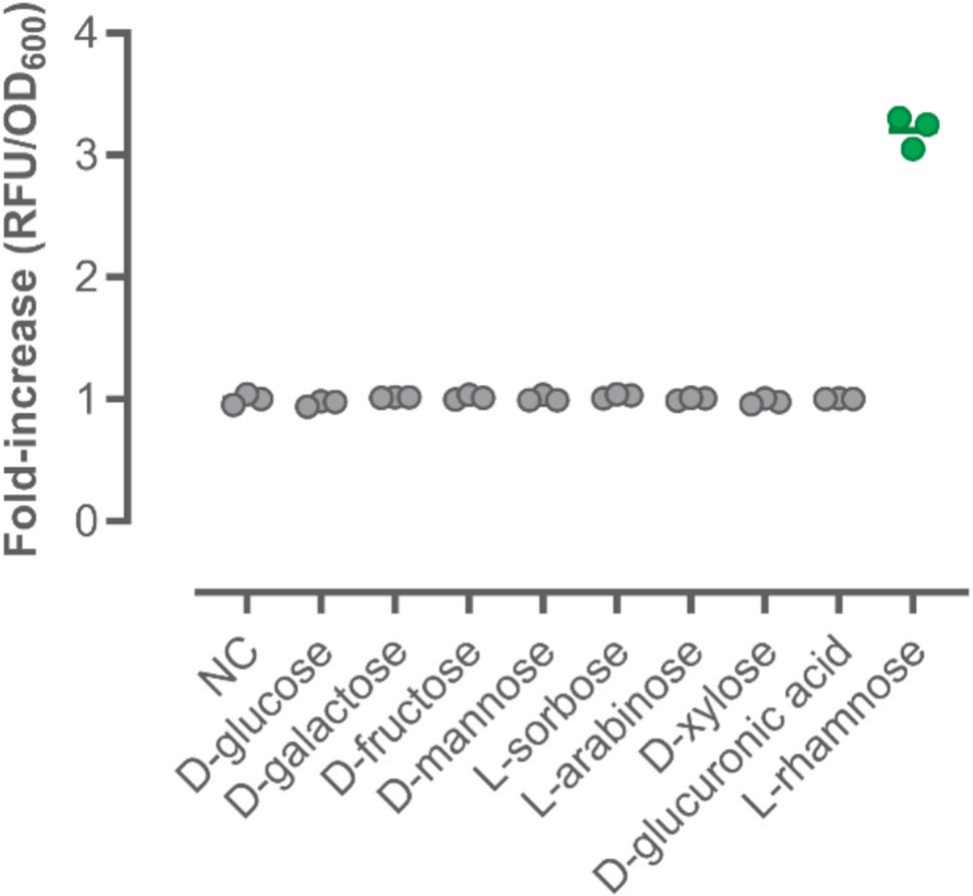
Response of the RhaS-based biosensor to various monosaccharides. Data shown as OD_600_-normalized fold-increase in fluorescence in pCK302-harboring cells after the addition of 100 µM of the indicated monosaccharides and cultivation at 37 °C for 4 h. Mean values and standard deviations were calculated from independent replicates (*n* = 3)

To reliably detect Rha in a complex mixture of oligo- and monosaccharides, for example, obtained from the enzymatic degradation of polysaccharide feedstocks, the specificity of the biosensor is particularly important. Therefore, other monosaccharides besides Rha were tested at a concentration of 100 µM for their induction potential. In addition to common sugars such as D-glucose, D-galactose, D-fructose, D-mannose, and L-sorbose, the monosaccharides L-arabinose, D-xylose, and D-glucuronic acid, which occur as building blocks in the complex polysaccharide ulvan, were investigated. Importantly, only Rha showed a three-fold increase in fluorescence, while the other monosaccharides did not yield fluorescence signals greater than the negative control (Fig. 1).

**Fig. 2:**
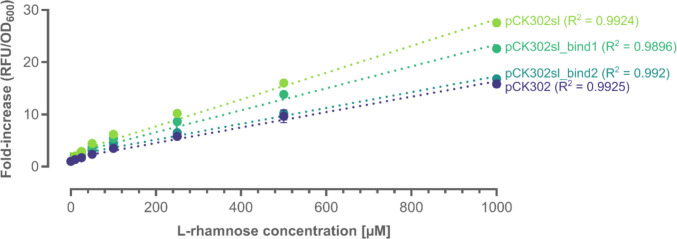
Genetic modifications of the biosensor system. Changes in the biosensor response upon introduction of a stem-loop upstream of the reporter gene (pCK302sl) and mutations in the activator binding sites (pCK302sl_bind1 and pCK302sl_bind2). The fold-increase in fluorescence from different constructs was monitored in response to increasing concentrations of Rha (0–1000 µM) after cultivation at 37 °C for 4 h. Mean values and standard deviations were calculated from independent replicates (*n* = 3)

To improve the operational window and sensitivity of Rha detection, different genetic variations in the region upstream of the open reading frame encoding the sfGFP reporter were introduced. The first featured the insertion of a 5′-stem loop (T7 g10 sl), which was reported to improve the stability of the mRNA, leading to the increased translation of target proteins (Mertens et al. 1996; Wegerer et al. 2008). Introduction of the stem loop yielded the plasmid pCK302sl (Table S1). The corresponding transformants exhibited almost two-fold improved fluorescence outputs over the range of tested Rha concentrations compared to pCK302 (Fig. 2). Motivated by this result, the two RhaS binding sites (i.e., half-sites 1 and 2) in proximity to the P_rha_BAD region were modified to improve binding of RhaS in the presence of the inducer Rha, thus enhancing reporter gene expression. Wickstrum and co-workers had shown that the DNA-binding domains of RhaS bound more tightly to the half-site 1 and that exchanging half-site 2 with a modified version of half-site 1, which does not disrupt the −35 region of P_rha_BAD, can lead to an increased induction (Wickstrum et al. 2007). Therefore, half-site 2 in pCK302sl was modified accordingly. To achieve this, the sequence of half-site 1, which is located on the reverse strand, was changed from GGCAACCAGGGAAAGAT to GGCAACCAGGGAAAGGT, yielding pCK302sl_bind1. The third biosensor construct, pCK302sl_bind2, had the half-site 2 sequence GTCAGTAACGAGAAGG, located on the forward strand, also replaced by GGCAACAGGCGAAAGGT; the underlined nucleotides represent the undisrupted −35 region of P_rha_BAD (Wickstrum et al. 2007). While pCK302sl_bind1 showed a slightly reduced fold-increase in fluorescence under experimental conditions compared to pCK302sl, the fluorescence output observed in pCK302sl_bind2 transformants was further reduced, showing a similar Rha-sensing behavior as the unmodified pCK302 parent (Fig. 2).

**Fig. 3:**
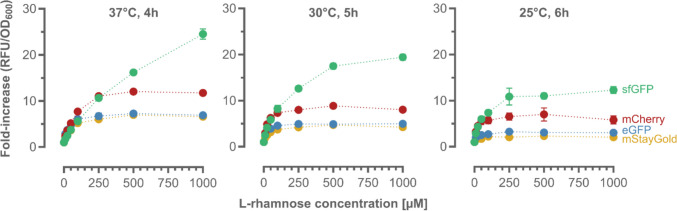
Implementation of different reporter genes. Changes in the biosensor response upon exchanging the reporter gene to different monomeric fluorescent proteins (sfGFP, eGFP, mStayGold, and mCherry) when cultivating transformants under the indicated conditions in the presence of Rha (0–1000 µM) after cultivation at 37 °C for 4 h. Mean values and standard deviations were calculated from independent replicates (*n* = 3)

So far, sfGFP has been used as the reporter gene (Pédelacq et al. 2006; Kelly et al. 2016). To examine the impact of different fluorescent proteins, enhanced GFP (eGFP) (Cormack et al. 1996), the green fluorescent monomeric StayGold (mStayGold) (Ando et al. 2024), and the red fluorescent mCherry (Shaner et al. 2004) were investigated (Bayer et al. 2023). Genes encoding the target reporter and the corresponding primers used for amplification and subsequent insertion into the pCK302sl backbone are given in Table S1. To consider varying maturation rates of the fluorescent proteins (Balleza et al. 2018; Guerra et al. 2022), different cultivation conditions (e.g., expression temperature and time) were investigated and the biosensor performance evaluated, based on the fold increase in fluorescence above background in response to varying Rha concentrations as before (Fig. 3).

Overall, the constructs with different reporter genes showed reduced fluorescence, independent of the cultivation conditions (Fig. 3). While the linear dose-response behavior of pCK302sl (featuring sfGFP as the reporter) covered the full concentration range of Rha (10–1000 µM), the operational window was limited (< 500 µM) for constructs featuring the alternative fluorescent proteins mCherry, eGFP, and mStayGold. Since the newly assembled pCK302sl construct performed best (Figs. 2 and 3), it was used for the biosensing of Rha in complex carbohydrate mixtures in the following.

**Fig. 4:**
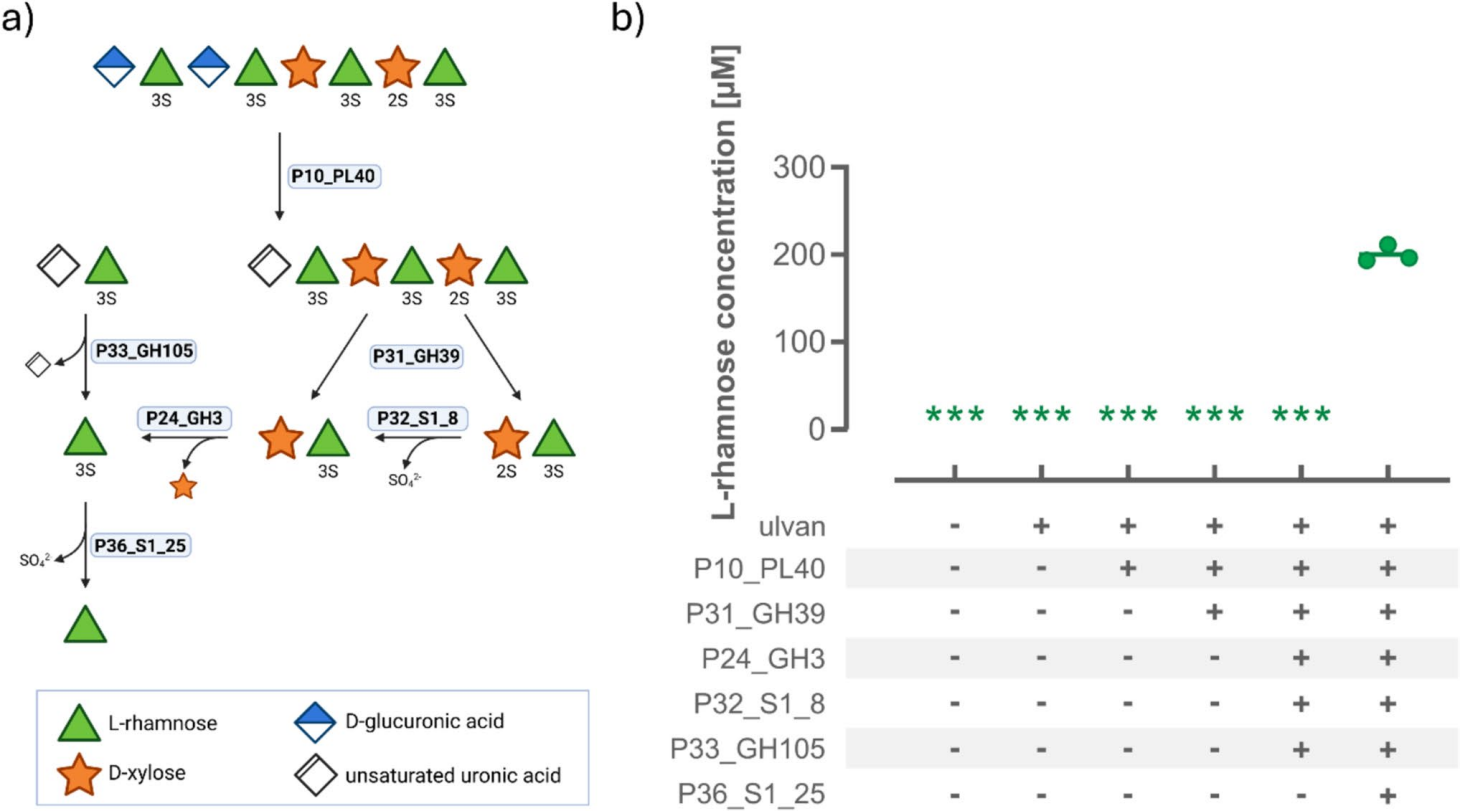
Biosensor-based monitoring of ulvan degradation. **a** The proposed degradation cascade of ulvan involves PUL H enzymes in *F. agariphila* (Reisky et al. 2019). Only a schematic fraction of the ulvan structure (top) is shown for clarity. The sulfation pattern is indicated by the letter S below the monosaccharides, including the number of the functionalized carbon. **b** The concentration of released Rha was determined by the biosensor, using a standard calibration (0–500 µM) recorded under the same experimental conditions. Whole-cell biosensor cultivation was performed at 37 °C for 4 h before measuring the fluorescence as before. Samples in which no Rha was detected are indicated by an asterisk. Mean values and standard deviations were calculated from independent replicates (*n* = 3)

The high complexity of algal polysaccharides requires microorganisms such as *F. agariphila* to coordinate the expression of multiple enzymes with complementary activities for metabolization. The genes encoding enzymes for the degradation of ulvan are located in the PUL H in *F. agariphila* KMM 3901^ T^ (Mann et al. 2013; Salinas and French 2017). However, depending on the polysaccharide’s composition, not all enzymes are essential for degradation; some even have redundant activities. Hence, only essential enzymes in the degradation cascade of ulvan to Rha were utilized in this work (Fig. 4a) (Reisky et al. 2019). In the initial step, an ulvan lyase (P10_PL40) cleaves the glycosidic bond between uronic acid units and Rha3S through an elimination reaction. This results in an unsaturated uronic acid at the non-reducing end, which can then be removed by an unsaturated glucuronyl hydrolase (P33_GH105), resulting in free Rha3S. If Xyl is present instead of a uronic acid, the xylosidase P31_GH39 can hydrolyze the bond between Xyl and Rha3S in the main chain. If the Xyl is sulfated as well, a sulfatase (P32_S1_8) removes the modification. To release Rha3S, the glycoside hydrolase P24_GH3 cuts the bond connecting Xyl and Rha3S in the corresponding disaccharide. Finally, the sulfatase P36_S1_25 desulfates Rha3S, releasing Rha, which is the most abundant monosaccharide in the complex polysaccharide ulvan from the marine *Ulva* species (Lahaye et al. 1997; Lahaye 1998; Lahaye and Robic 2007). Therefore, Rha is well-suited for use as an effective marker for the complete depolymerization of ulvan. Various stages of the ulvan degradation cascade were simulated using the indicated PUL H enzymes to determine whether intermediate ulvan degradation products interfered with the biosensor-based detection of the target Rha.

As expected, controls without polysaccharides or enzymes showed no fold-increase in fluorescence, indicating the absence of free Rha (Fig. 4b). Across all intermediate stages of ulvan degradation, involving ulvan lyase, GH39, GH3, S1_8, and GH105, no release of Rha was detected according to biosensor-based measurements. Only after adding the sulfatase S1_25 did the biosensor yield a fluorescence signal corresponding to approximately 200 µM Rha, as determined by the standard dilution series measured simultaneously. Importantly, these results agreed with the Rha concentration determined using a commercial kit from Megazyme (Figure S2).

**Fig. 5:**
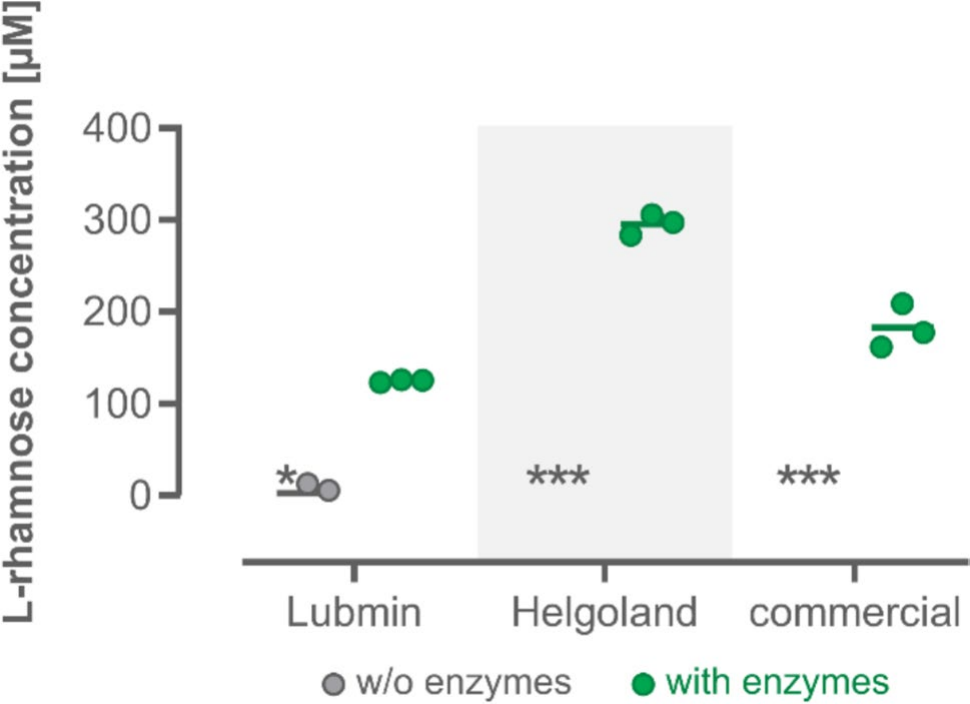
Enzymatic treatment of algal biomass samples. Each algal sample was incubated with all important PUL H enzymes (see Fig. 4). The concentration of released Rha was determined by the biosensor, using a standard calibration (0–500 µM Rha) recorded under the same experimental conditions. Cultivations were performed at 37 °C for 4 h as before. Samples in which no Rha was detected are indicated by an asterisk. Mean values and standard deviations were calculated from independent replicates (*n* = 3)

Although *Ulva* sp. macroalgae contain a high proportion of the polysaccharide ulvan (Lahaye and Robic 2007), its extraction is an additional time-consuming step (Carvalho et al. 2024). Therefore, it was investigated whether a direct degradation of the polysaccharide from dried algal biomass is possible. Three different algal samples were used for this purpose. Two samples were collected from two different beaches along the Baltic Sea (Lubmin) and the North Sea (Helgoland) in Germany. The third sample was a commercially available sample from an *Ulva* species. No Rha release was detected in the absence of enzymes in any of the three biomass samples employed. When the full enzyme cocktail was added as for the degradation of ulvan (Fig. 4), Rha was released according to the biosensor-based monitoring as well as using the commercial kit (Fig. 5 and Figure S3, respectively). While both detection methods indicated the highest Rha concentration in samples from the degradation of the algae biomass from Helgoland, deviations in the measured Rha concentrations (286 ± 8.9 µM and 537 ± 17.2 µM, respectively) may be explained by the release of pigments from the biomass, interfering with the absorbance-based quantification of Rha by the commercial kit.

## Discussion

The accumulation of massive quantities of algal biomass is a growing problem due to rising ocean temperatures and nutrient levels from global warming and intensive agriculture, respectively. However, the polysaccharides contained in algal biomass represent a widely unexploited source of rare sugars and a cheap and renewable resource for biotechnological applications. One of the obstacles to accelerating the utilization of this waste feedstock is the lack of inexpensive yet accurate analytical methods to assess both the activity of enzymes involved in the depolymerization in high-throughput and the quantification of released monosaccharides. To date, the analysis of polysaccharide degradation has mostly been based on chromatographic methods or through the quantification of newly formed reducing ends. These methods may involve laborious sample preparation and are time-consuming and low-throughput. Furthermore, only limited information about the actual degradation products is provided by reducing-end assays.

To address these shortcomings, a genetically encoded biosensor based on the RhaS transcription factor and its cognate P_rha_BAD promoter from *E. coli* and fluorescent reporter proteins was developed in this study for the robust detection of the monosaccharide Rha. The latter is a major constituent of the complex polysaccharide ulvan, found in substantial amounts in algal biomass waste. Implementation of an mRNA-stabilizing stem loop (T7 g10 sl) in the 5′-untranslated region of the sfGFP-encoding reporter gene, yielding pCK302sl, significantly improved the signal-to-noise ratio of the fluorescence output, while conserving the specificity for Rha (Fig. 1) and a broad operational window of the biosensor (Fig. 2), allowing for the quantification of the monosaccharide from 10 µM to 1 mM (Figure S1), which is superior to previously reported biosensor systems for Rha (Lukasiak et al. 2012; Kelly et al. 2016). Modifying either of the two RhaS binding sites according to Wickstrum et al*.*, yielding pCK302sl_bind1 and pCK302sl_bind2 (Fig. 2), or exchanging sfGFP for alternative fluorescent reporters (eGFP, mStayGold, mCherry) and adapting cultivation conditions (Fig. 3) did not further improve biosensor performance (Cardinale and Arkin 2012).

Ultimately, the pCK302sl-based whole-cell biosensor system was successfully applied to monitor the biodegradation of the marine polysaccharide ulvan by the activities of six purified enzymes from PUL H of *F. agariphila*. The release of the monosaccharide Rha depended strictly on the sulfatase P36_S_1_25. This not only suggests that the biosensor is a suitable tool to study metabolic pathways, such as the degradation of ulvan (Fig. 4a) (Reisky et al. 2019), but also highlights the capability of the RhaS-based biosensor to exclusively detect free Rha in complex mixtures containing other (functionalized) mono- and oligosaccharides (Fig. 4b). Importantly, the biosensor-based quantification of Rha correlated with that of a commercial kit. The biosensor was also employed to monitor Rha release from shredded but otherwise unpretreated biomass of three different algae samples of *Ulva* species and can certainly be extended to other (algal-based) feedstocks (Sharma and Horn 2016; Teune et al. 2025). In this study, the enzymatic treatment of a specimen from the North Sea (Helgoland, Germany) released the highest concentration of Rha. Deviations between biological samples may be explained by varying total amounts of polysaccharides and different compositions of ulvan (e.g., higher content of Xyl instead of D-glucuronic acid or additional ß-1,2-linked sugars) (Reisky et al. 2019).

In comparison with other well-established quantification methods for L-rhamnose, the limit of detection (LOD) of the biosensor system represents a current constraint. For example, a LOD as low as 50 nM for monosaccharides in an HPAEC-PAD (high-performance anion-exchange chromatography with pulsed amperometric detection) has been demonstrated (Nouara et al. 2019), whereas a LOD < 3.0 µM can be achieved by electrochemical sensors in complex matrices (Patella et al. 2025). An enzymatic assay kit based on L-rhamnose dehydrogenase (K-RHMANOSE, Megazyme) achieves a limit of detection of 7 µM of Rha, comparable to the transcription factor-based biosensor presented in this study. Another limitation may be the presence of substances in the samples to be tested that severely impair the growth of *E. coli* cells, which can be partly addressed by normalizing fluorescence signals to optical density.

To increase biosensor sensitivity and lower the LOD, for example, genetic engineering of the promoter region or tuning of the ribosome binding site (of the reporter) could be implemented—in addition to the improvements to the biosensor system already achieved in this study. Furthermore, targeted protein engineering of the ligand-binding domain of the regulatory protein RhaS or recombinant overexpression of the rhamnose transporter RhaT (Baldomá et al. 1990) to overcome catabolite repression by glucose (Fox and Prather 2020) and to increase intracellular Rha concentrations in *E. coli* biosensor cells may enhance biosensor sensitivity. These methods have been successfully employed to enhance the performance for various other genetically encoded biosensor systems (Bayer et al. 2023).

Since polysaccharides from algal biomass, such as ulvan, are a renewable and ubiquitous source of Rha, the developed Rha biosensor could serve to optimize enzymatic depolymerization. In a high-throughput process, this method could be used to optimize reaction conditions and perform protein engineering of the involved enzymes. The same applies to the industrially relevant decomposition of the bittering agent naringin in citrus juices (Li et al. 2018).

Together, the refined biosensor in this study expanded its application from a simple recombinant expression system (Krebsfänger et al. 1998; Wegerer et al. 2008; Kelly et al. 2016) to the analysis of the multi-enzyme degradation of complex polysaccharides such as ulvan with improved sensitivity compared to previously reported RhaS-based systems (Lukasiak et al. 2012) and a broad operational window. While the biosensor system investigated in this study represents a simple and robust analytical tool, independent of established chromatographic or spectrophotometric methods, for the detection and quantification of Rha in complex matrices, the general biosensing strategy may not only be applied to other mono- and polysaccharides. It offers immense potential to assist in the functional assessment of PULs, accelerate the engineering of carbohydrate-active and related enzymes (Li et al. 2018), and optimize reaction conditions for enzymatic depolymerization for the efficient valorization of renewable feedstocks for versatile industrial applications in the future.

## Supplementary Information

Below is the link to the electronic supplementary material.

ESM 1 (2.13 MB PDF)

## Data Availability

The datasets generated and/or analyzed during the current study are available from the corresponding author upon reasonable request.
