# Supplementary material for: A genetically encoded L-rhamnose biosensor for monitoring marine polysaccharide depolymerization
Source: Appl Microbiol Biotechnol. 2026 Jan 29;110:50. doi: 10.1007/s00253-026-13724-1 (PMC12858550; doi:10.1007/s00253-026-13724-1)
Supplement: Supplementary file 1 [file 253_2026_13724_MOESM1_ESM.pdf]

Supplementary Information

A genetically encoded L-rhamnose biosensor for monitoring marine polysaccharide depolymerization

Yannick Wolf[1], Thomas Bayer[1], and Uwe T. Bornscheuer[1]*

[1]Institute of Biochemistry, Department of Biotechnology and Enzyme Catalysis, University of Greifswald, Greifswald, Germany

*Corresponding author: E-mail: uwe.bornscheuer@uni-greifswald.de

Amino acid sequences of the used fluorescence proteins

superfolderGFP (green fluorescence protein from *Aequorea victoria*, GenBank accession code: PX636966):

MRKGEELFTGVVPILVELDGDVNGHKFSVRGEGEGDATNGKLTLKFICTTGKLPVPWPTLVT
TLTYGVQCFARYPDHMKQHDFFKSAMPEGYVQERTISFKDDGTYKTRAEVKFEGDTLVNRIE
LKGIDFKEDGNILGHKLEYNFNSHNVYITADKQKNGIKANFKIRHNVEDGSVQLADHYQQNT
PIGDGPVLLPDNHYLSTQSVLSKDPNEKRDHMVLLEFVTAAGITHGMDELYKGSGSGSHHHH
HH

enhancedGFP (red fluorescence protein from *Aequorea Victoria*, GenBank accession code: PX636967):

MVSKGEELFTGVVPILVELDGDVNGHKFSVSGEGEGDATYGKLTLKFICTTGKLPVPWPTLV
TTLTYGVQCFSRYPDHMKQHDFFKSAMPEGYVQERTIFFKDDGNYKTRAEVKFEGDTLVNRI
ELKGIDFKEDGNILGHKLEYNYNSHNVYIMADKQKNGIKVNFKIRHNIEDGSVQLADHYQQN
TPIGDGPVLLPDNHYLSTQSALSKDPNEKRDHMVLLEFVTAAGITLGMDELYK

mCherry (red fluorescence protein from *Discosoma* sp., GenBank accession code: PX636968):

MVSKGEEDNMAIIKEFMRFKVHMEGSVNGHEFEIEGEGEGRPYEGTQTAKLKVTKGGPLPFA
WDILSPQFMYGSKAYVKHPADIPDYLKLSFPEGFKWERVMNFEDGGVVTVTQDSSLQDGEFI
YKVKLRGTNFPSDGPVMQKKTMGWEASSERMYPEDGALKGEIKQRLKLKDGGHYDAEVKT
TYKAKKPVQLPGAYNVNIKLDITSHNEDYTIVEQYERAEGRHSTGGMDELYK

mStayGold (red fluorescence protein from *Cytaeis uchidae*, GenBank accession code: PX636969):

MVSTGEELFTGVVPFKFQLKGTINGKSFTVEGEGEGNSHEGSHKGKYVCTSGKLPMSWAALG
TSFGYGMKYYTKYPSGLKNWFHEVMPEGFTYDRHIQYKGDGSIHAKHQHFMKNGTYHNIVE
FTGQDFKENSPVLTGDMDVSLPNEVQHIPIDDGVECTVTLQYPLLSDESKCVEAYQNTIIKPLH
NQPAPDVPFHWIRKQYTQSKDDTEERDHIIQSETLEAHL

Nucleotide sequences of the region upstream of the reporter gene (between M13 site and start codon)

pCK302 (original plasmid sequence without modifications):

gtaaaacgacggccagtcttaagctcgggccccctgggcggttctgataacgagtaatcgttaatccgcaaataacgtaaaaacccgcttcggcgggtttttttatgggggggagtttagggaaagagcatttgtcagaatatttaagggcgcctgtcactttgcttgatatatgagaattatttaaccttataaatgagaaaaaagcaacgcactttaaataagatacgttgctttttcgattgatgaacacctataattaaactattcatctattatttatgattttttgtatatacaatatttctagtttgttaaagagaattaagaaaataaatctcgaaaataataaagggaaaatcagtttttgatatcaaaattatacatgtcaacgataatacaaaatataatacaaactataagatgttatcagtatttattatgcatttagaataaattttgtgtcgcccttccacaattcagcaaattgtgaacatcatcacgttcatctttccctggttgccaatggcccattttcctgtcagtaacgagaaggtcgcgtattcaggcgctttttagactggtcgtaatgaaacggtttccctctagaaataatttgtttaactttaggaggtaaaacat

pCK302sl (plasmid with an introduced T7 g10 stem-loop):

gtaaaacgacggccagtcttaagctcgggccccctgggcggttctgataacgagtaatcgttaatccgcaaataacgtaaaaacccgcttcggcgggtttttttatgggggggagtttagggaaagagcatttgtcagaatatttaagggcgcctgtcactttgcttgatatatgagaattatttaaccttataaatgagaaaaaagcaacgcactttaaataagatacgttgctttttcgattgatgaacacctataattaaactattcatctattatttatgattttttgtatatacaatatttctagtttgttaaagagaattaagaaaataaatctcgaaaataataaagggaaaatcagtttttgatatcaaaattatacatgtcaacgataatacaaaatataatacaaactataagatgttatcagtatttattatgcatttagaataaattttgtgtcgcccttccacaattcagcaaattgtgaacatcatcacgttcatctttccctggttgccaatggcccattttcctgtcagtaacgagaaggtcgcgtattcaggcgctttttagactggtcgtagggagaccacaacggtttccctctagaaataattttcttaagaaggagatatacat

pCK302sl_bind1 (plasmid with an introduced T7 g10 stem-loop and modified RhaS binding site 1):

gtaaaacgacggccagtcttaagctcgggccccctgggcggttctgataacgagtaatcgttaatccgcaaataacgtaaaaacccgcttcggcgggtttttttatgggggggagtttagggaaagagcatttgtcagaatatttaagggcgcctgtcactttgcttgatatatgagaattatttaaccttataaatgagaaaaaagcaacgcactttaaataagatacgttgctttttcgattgatgaacacctataattaaactattcatctattatttatgattttttgtatatacaatatttctagtttgttaaagagaattaagaaaataaatctcgaaaataataaagggaaaatcagtttttgatatcaaaattatacatgtcaacgataatacaaaatataatacaaactataagatgttatcagtatttattatgcatttagaataaattttgtgtcgcccttccacaattcagcaaattgtgaacatcatcacgttcacctttccctggttgccaatggcccattttcctgtcagtaacgagaaggtcgcgtattcaggcgctttttagactggtcgtagggagaccacaacggtttccctctagaaataattttcttaagaaggagatatacat

pCK302sl_bind2 (plasmid with an introduced T7 g10 stem-loop and modified RhaS binding site 2):

gtaaaacgacggccagtcttaagctcgggccccctgggcggttctgataacgagtaatcgttaatccgcaaataacgtaaaaacccgcttcggcgggtttttttatgggggggagtttagggaaagagcatttgtcagaatatttaagggcgcctgtcactttgcttgatatatgagaattatttaaccttataaatgagaaaaaagcaacgcactttaaataagatacgttgctttttcgattgatgaacacctataattaaactattcatctattatttatgattttttgtatatacaatatttctagtttgttaaagagaattaagaaaataaatctcgaaaataataaagggaaaatcagtttttgatatcaaaattatacatgtcaacgataatacaaaatataatacaaactataagatgttatcagtatttattatgcatttagaataaattttgtgtcgcccttccacaattcagcaaattgtgaacatcatcacgttcatctttccctggttgccaatggcccattttcctggcaacaggcgaaaggtcgcgtattcaggcgctttttagactggtcgtagggagaccacaacggtttccctctagaaataattttcttaagaaggagatatacat

Table S1 List of plasmids and primers. Each plasmid and primer is described by name, brief description or sequence, and origin.

**Name of plasmid or primer**	**Description**	**Origin**
**Plasmids**		
pCK302	pJ404 containing *E. coli* rha_{BAD} promoter upstream of sfGFP, rhaS downstream of ampR	(Kelly et al. 2016)
pCK302sl	pCK302 containing a T7 g10 stem-loop between the rha_{BAD} promoter region and sfGPF	This study
pCK302sl_bind1	pCK302sl with a modified rhaS binding site 1	This study
pCK302sl_bind2	pCK302sl with a modified rhaS binding site 2	This study
pCK302sl_mCherry	pCK302sl carrying mCherry downstream of rha_{BAD} promoter instead of sfGFP	This study
pCK302sl_eGFP	pCK302sl carrying eGFP downstream of rha_{BAD} promoter instead of sfGFP	This study
pCK302sl_mStayGold	pCK302sl carrying mStayGold downstream of rha_{BAD} promoter instead of sfGFP	This study
**Primers**		
pCK302sl_fw	gaaataattttcttaagaaggagatatacatatgcgtaaaggcg	This study
pCK302sl_rv	tagagggaaaccgttgtggtctccctacgaccagtctaaaaagc	This study
pCK302_RhaS_bind1_fw	cctttccctggttgccaatg	This study
pCK302_RhaS_bind1_rv	tgaacgtgatgatgttcacaatttg	This study
pCK302_RhaS_bind2_fw	gcgaaaggtcgcgtattcaggcgc	This study
pCK302_RhaS_bind2_rv	ctgttgccaggaaaatgggccattgg	This study
pCK302sl_mC_fw	gatgaactgtataaataactcgagccccaaggg	This study
pCK302sl_mC_rv	cacctttgctcaccatcatatgtatatctccttcttaagaaaattatttctagaggg	This study
mC_pCK302sl_fw	gaaggagatatacatatggtgagcaaaggtgaagaagataatatg	This study
mC_pCK302sl_rv	cagatgcgcctccaaggtttctttatacagttcatccataccaccg	This study
pCK302sl_eGFP_fw	ggatgaactgtataaataactcgagccccaaggg	This study
pCK302sl_eGFP_rv	cgccccttactaaccatatgtatatctccttcttaagaaaattatttctagaggg	This study
eGFP_pCK302sl_fw	aagaaggagatatacatatggttagtaagggcgaagaactgttcac	This study
eGFP_pCK302sl_rv	ttggggctcgagttatttatacagttcatccatgcccagg	This study
pCK302sl_mSG_fw	ttggaggcgcatctgtaactcgagccccaaggg	This study
pCK302sl_mSG_rv	tcctgttgataccatatgtatatctccttcttaagaaaattatttctagaggg	This study
mSG_pCK302sl_fw	gaaggagatatacatatggtatcaacaggagaagagctatttac	This study
mSG_pCK302sl_rv	ttggggctcgagttacagatgcgcctccaaggtttc	This study

Figure S1:

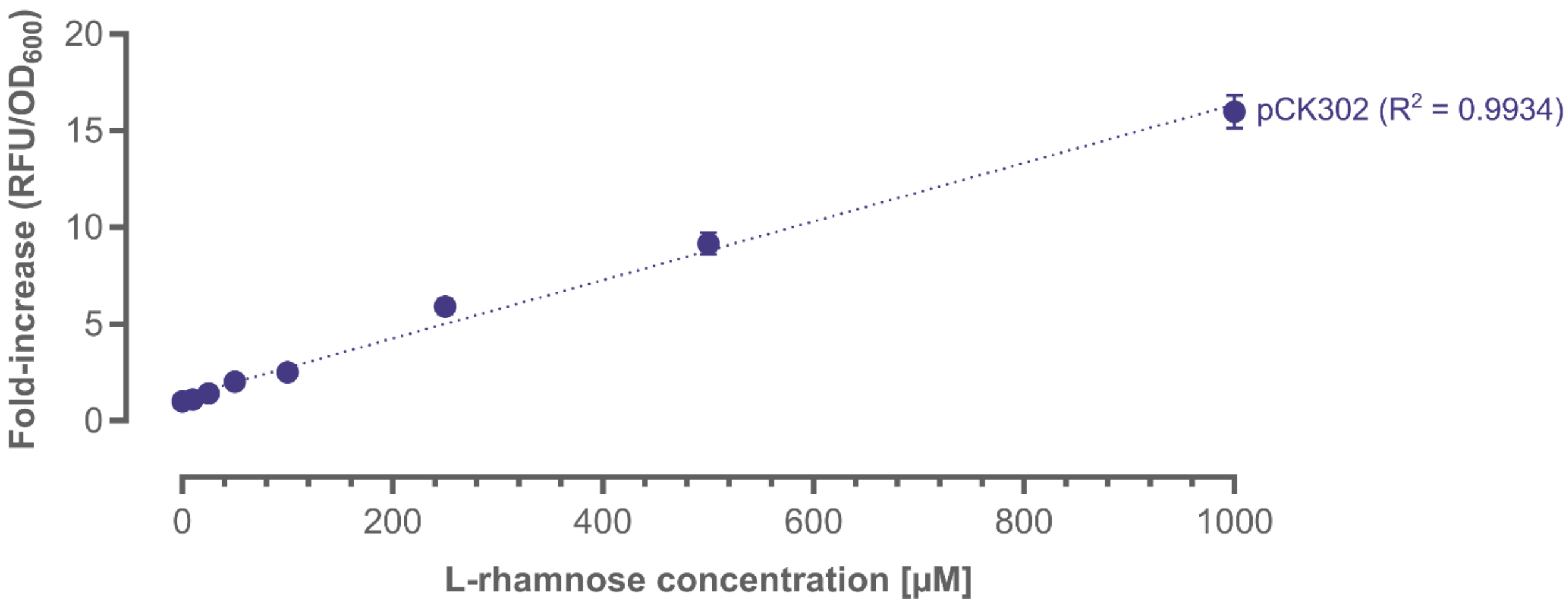

Figure S1: Fold-increase of the fluorescence divided by the optical density using the biosensor on increasing L-rhamnose concentrations (0–1000 µM) after 4 hours biosensor cultivation at 37 °C. Mean values and standard deviations were calculated from independent replicates (n = 3).

Figure S2:

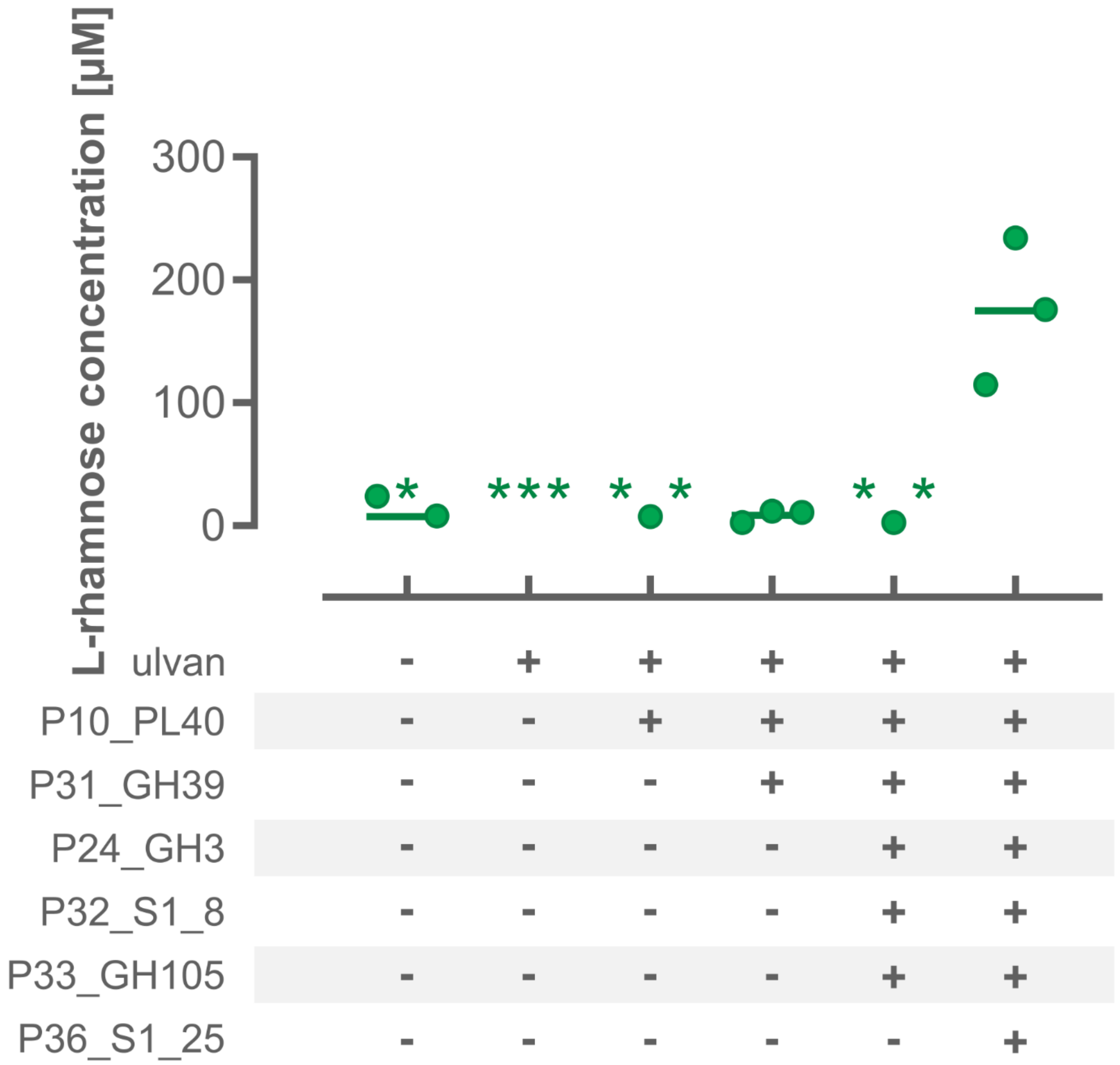

Figure S2: Following the degradation of the extracted marine polysaccharide ulvan. The concentration of released Rha was determined by the L-rhamnose assay kit from Megazyme. Samples in which no Rha was detected are indicated by an asterisk. Mean values and standard deviations were calculated from independent replicates (n = 3).

Figure S3:

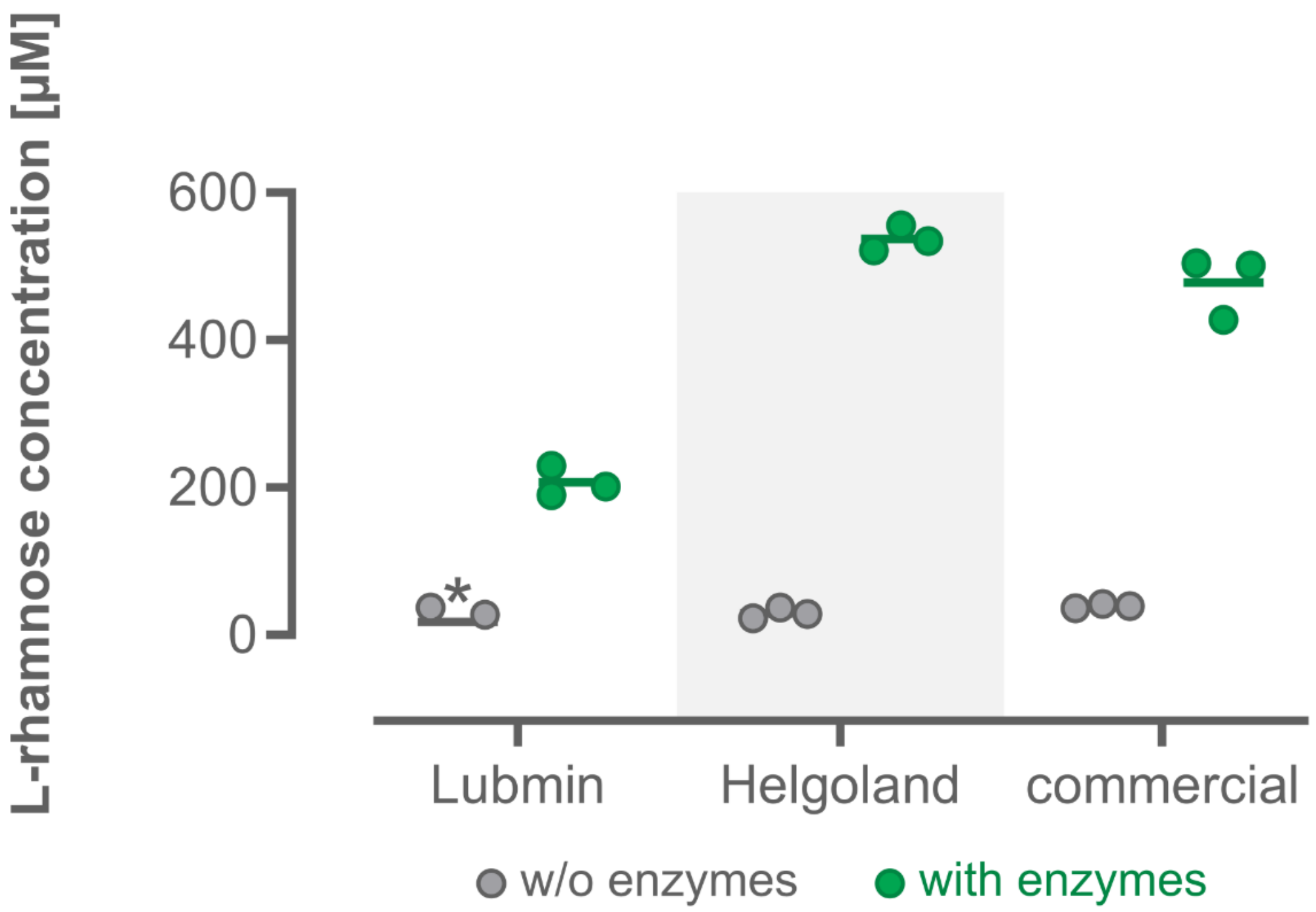

Figure S3: Enzymatic treatment of algal biomass samples. Each algal sample was incubated with all important PUL H enzymes (see Figure 4). The concentration of released Rha was determined by the L-rhamnose assay kit from Megazyme. Samples in which no Rha was detected are indicated by an asterisk. Mean values and standard deviations were calculated from independent replicates (n = 3).

Figure S4:

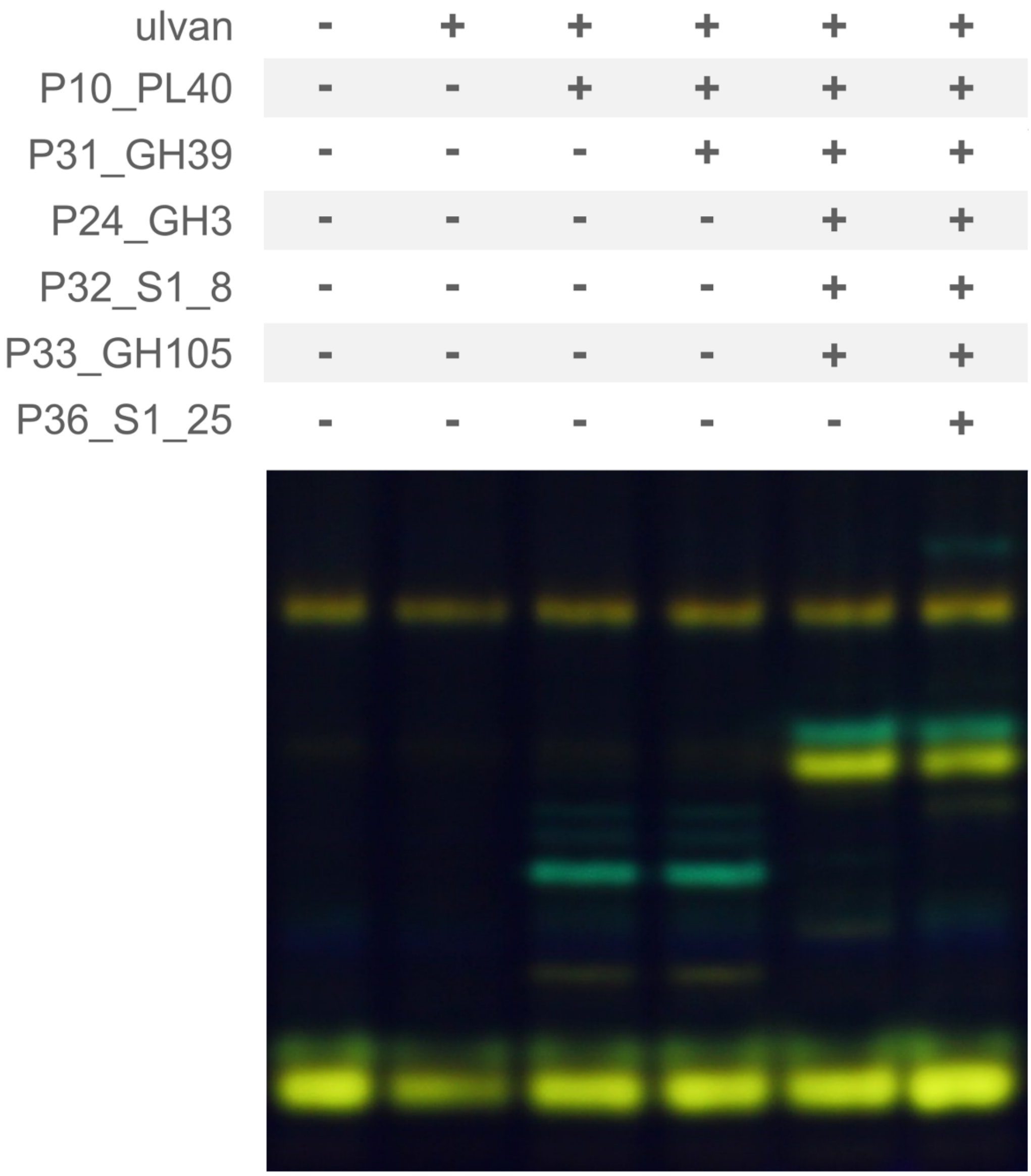

Figure S4: Monitoring the formation of saccharide products during the degradation of the extracted marine polysaccharide ulvan. Based on the AMAC FACE gel shown here, the product formation of the various stages of the enzymatic degradation cascade can be observed as fluorescent bands between the background bands caused by the dye above and below.

Figure S5:

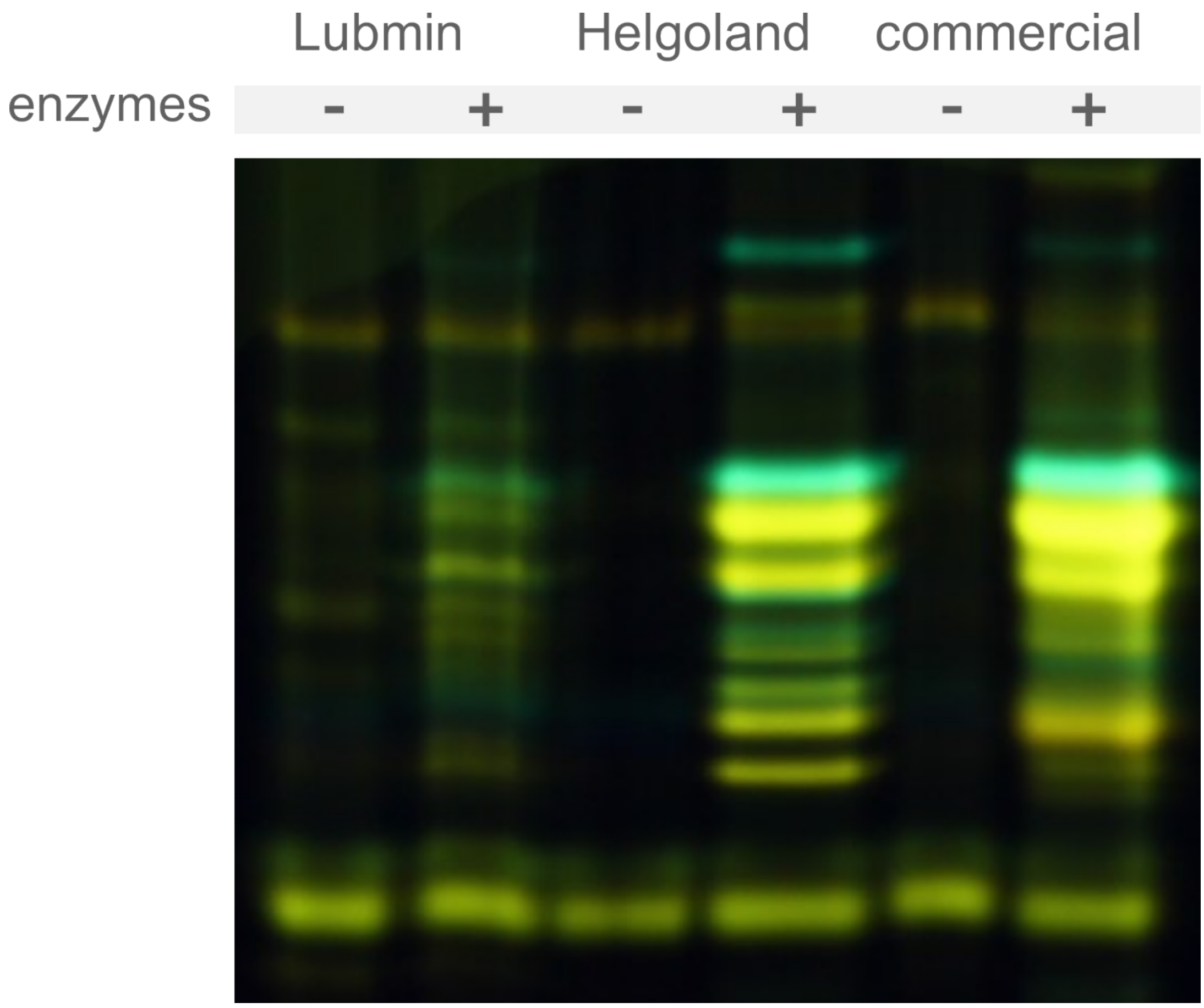

Figure S5: Monitoring the formation of saccharide products during the degradation of various Ulva sp. biomass samples. Based on the AMAC FACE gel shown here, the product formation of the various stages of the enzymatic degradation cascade can be observed as fluorescent bands between the background bands caused by the dye above and below.
